# Dynamic Changes in AQP4-IgG Level and Immunological Markers During Protein-A Immunoadsorption Therapy for NMOSD: A Case Report and Literature Review

**DOI:** 10.3389/fimmu.2021.650782

**Published:** 2021-07-21

**Authors:** Bo Chen, Chuan Qin, Man Chen, Hai-Han Yu, Ran Tao, Yun-Hui Chu, Bi-Tao Bu, Dai-Shi Tian

**Affiliations:** Department of Neurology, Tongji Hospital, Tongji Medical College, Huazhong University of Science and Technology, Wuhan, China

**Keywords:** protein-A immunoadsorption, rescue therapy, AQP4-IgG, neuromyelitis optica spectrum disorder, case report

## Abstract

The changes in the serum levels of aquaporin-4-IgG (AQP4-IgG), immunoglobulins, and inflammatory mediators in neuromyelitis optica spectrum disorder (NMOSD) cases treated with immunoadsorption have been rarely described in detail. Here we report a 29-year-old steroid-resistant NMOSD female with a severe disability (bilateral blindness and paraplegia) who received protein-A immunoadsorption as a rescue treatment. During the total 5 sessions, the circulating level of AQP4-IgG, immunoglobulins, and complement proteins (C3 and C4) showed a rapid and sawtooth-like decrease, and the serum AQP4-IgG titer declined from 1:320 to below the detectable limit at the end of the 3rd procedure. Of all the antibodies, IgG had the biggest removal rate (>96.1%), followed by IgM (>66.7%) and IgA (53%), while complement C3 and C4 also dropped by 73% and 65%, respectively. The reduced pro-inflammatory cytokines (interleukin-8 and tumor necrosis factor-α) and marked increased lymphocyte (T and B cell) counts were also observed. The improvement of symptoms initiated after the last session, with a low AQP4-IgG titer (1:32) persisting thereafter. Accordingly, protein-A immunoadsorption treatment could be one of the potential rescue therapies for steroid-resistant NMOSD patients with a severe disability.

## Introduction

Neuromyelitis optica spectrum disorder (NMOSD) is commonly considered an antibody-mediated autoimmune debilitating disease, with only a small proportion of acute NMOSD attacks achieving a complete remission (1, 2). The pathogenic aquaporin-4-IgG (AQP4-IgG) can be detected in most NMOSD patients and tends to be associated with frequent relapses (3, 4). Traditionally, the majority of the sufferers can benefit from pulsed high-dose intravenous methylprednisone (IVMP), accelerating clinical improvement and shortening the acute phase. For those refractory patients with severe attacks who are insufficiently responsive to glucocorticoids, plasma exchange (PE) and immunoadsorption can be alternative rescue or adjunctive therapies. In fact, previous studies have noted that the apheresis techniques, especially used as the first-line therapy in the early stage, can achieve a better outcome than steroids in treating NMOSD attacks (5, 6). Compared to glucocorticoids, this treatment strategy seems to exert a quicker and more potent effect on controlling the excessive immune response *via* direct removal of antibodies, pro-inflammatory cytokines, and complement proteins from the serum, which might reduce the relapse in a short term due to the subsequent reduced serum pathogenic AQP4-IgG concentration.

Nevertheless, the potential exposure to blood-borne diseases, allergens, and the shortage of plasma limit its wide application. Theoretically, protein-A immunoadsorption can selectively remove immunoglobulins and complement proteins without transfusing foreign blood products, spare albumin, and clotting factors, and have fewer adverse effects, which appear to be superior to PE (7–9). Previous reports have mentioned the changes of the antibody levels in patients with AQP4-IgG seropositive NMOSD during the immunoadsorption (8, 10), without involving the complement proteins, cytokine profiles, and lymphocyte system. Here, we report a case with a severe disability due to NMOSD relapse recovered by protein-A immunoadsorption, with a detailed description of the alteration in serum AQP4-IgG titer, the concentration of inflammatory mediators, and the lymphocyte subsets.

## Case Presentation

**Figure 1:**
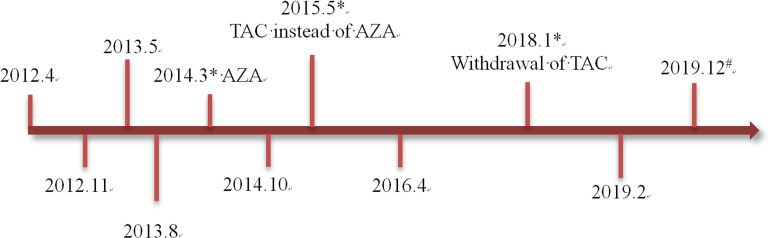
The timeline with relevant data of the past episodes and interventions. *Severe relapse treated with plasma exchange. ^#^This admission. AZA, azathioprine; TAC, tacrolimus.

A 29-year-old female who was first diagnosed with NMOSD in 2012 could recover from pulsed glucocorticoids during the initial several attacks. However, since 2014, she benefited little from this therapy and began to receive PE as the rescue treatment during the 3 severe relapses (Expanded Disability Status Scale, EDSS score ≥6) in the following 5 years. *Azathioprine (150mg/d for 1 year)* and t*acrolimus (3mg/d for 2.5 years)* were given as the maintenance therapies, respectively, but failed to prevent the clinical attacks. The detailed timeline with relevant data of the past episodes and interventions was summarized in **Figure 1**. Three days before admission, she suffered paraplegia and blindness without any immunosuppressant treatment. No other personal or family history of autoimmune diseases was reported. Drug abuse and psychological disorders were denied, either. Owing to the occurrence of the ongoing severe disability and lack of plasma, protein-A immunoadsorption was tried with consent from the patient.

**Figure 2:**
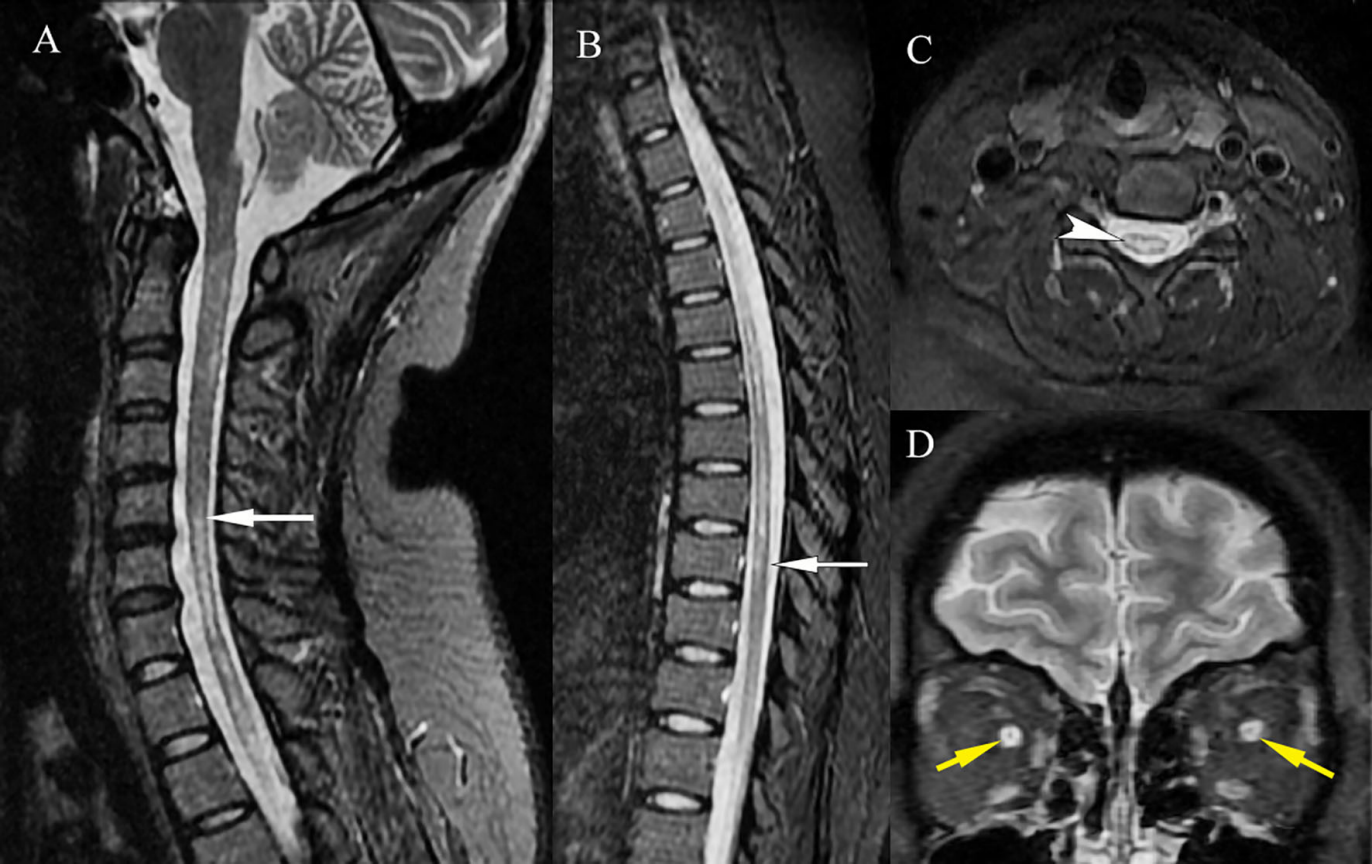
MRI of the NMOSD lesions. MRI of the cervical and thoracic spine showed a longitudinally extensive T2-hyperintense lesion [**(A, B)**, white arrows], which involved the central portion of the cord [**(C)**, white arrowhead]. A significant enhancement and thickening of the bilateral optic nerve sheaths were observed [**(D)**, yellow arrows].

At nadir, neurological examination revealed paraplegia, with hypermyotonia and tendon hyperreflexia. She also had bilateral blindness without light perception, and her EDSS score was assessed at 8. MRI of the cervical and thoracic spine showed a longitudinally extensive T2-hyperintense lesion, with the central portion of the cord involved (**Figures 2A–C**). A significant enhancement and thickening of the optic nerve sheaths were also observed (**Figure 2D**).

**Figure 3:**
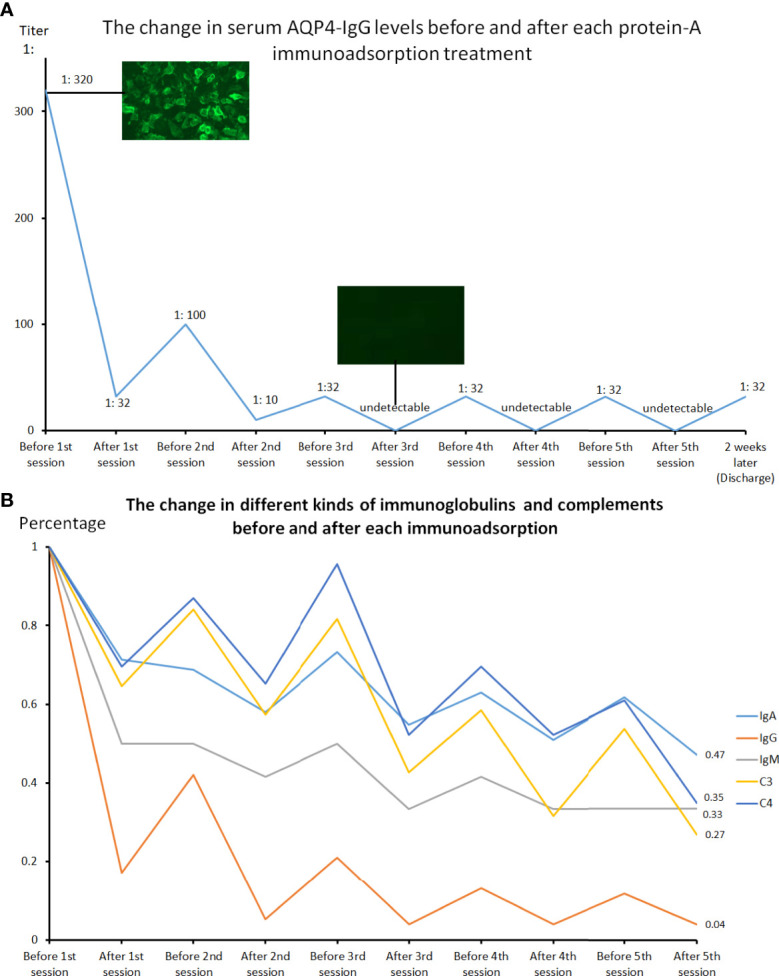
The changes in serum AQP4-IgG levels performed by cell-based assay before and after each protein-A immunoadsorption treatment **(A)** and the changes in serum concentration of immunoglobulins and complement proteins before and after each session **(B)**. Each treatment could lead to a significant decline in the serum AQP4-IgG titer, IgA, IgG, IgM, as well as C3 and C4 levels, with a slight rebound before the next therapy.

The complete blood cell count, basic metabolic panel, and liver function were within normal limits. A cerebral spinal fluid (CSF) study showed a normal cell count (0*10^6^/L, reference range: 0-8*10^6^/L), protein level, and oligoclonal band, with an IgG index of 0.6 (reference range: 0-0.7). AQP4-IgG tested by cell-based assay (CBA) revealed a positive result, with a titer of 1:320 in the serum (**Figure 3A**) and 1:1 in the CSF, while the myelin oligodendrocyte glycoprotein antibody (MOG-IgG), glial fibrillary acidic protein antibody (GFAP-IgG), myelin basic protein antibody (MBP-IgG), AQP1-IgG, and Flottilin1/2-IgG in the serum and CSF, which were also measured by CBA, were undetectable.

The patient consent was then obtained before intravenous catheterization and combined anticoagulation with heparin. The protein-A immunoadsorption was started one week after onset, with the pulsed glucocorticoids (1000mg/d) and concomitant supportive therapies given 3 days ago. The immunoadsorption column (KONPIA, KONCEN, China) can be reused no more than 10 sessions as long as the absorbed antibodies are eluted from the column after each procedure according to the product instruction, while the plasma separator and tubing system are for single use only. During 5 sessions, each treatment filtered approximately 3 liters of plasma every other day. The levels of AQP4-IgG, complement proteins (C3 and C4), and immunoglobulins in the serum were detected at the beginning and the end of every procedure. Cytokine profiles (solid-phase two-site chemiluminescent immunometric assay, IMMULITE 1000 Analyzer, Siemens) and lymphocyte subsets (flow cytometry, BD Biosciences) were only analyzed before and after all the sessions. The results revealed that each treatment could lead to a significant reduction in the AQP4-IgG titer, while a slight rebound was always observed before the start of the next therapy (**Figure 3A**). The serum AQP4-IgG decreased rapidly to below the detectable limit after the 3rd session of treatment and kept a lower titer (1:32) until the patient was discharged 2 weeks later. The IgG, IgA, IgM, C3, and C4 demonstrated almost the same trends (**Figure 3B**). Of all the antibodies, IgG had the biggest reduction rate (>96.1%), followed by IgM (>66.7%), and IgA was least able to be eliminated (53%) (**Table 1**). Interestingly, C3 and C4 components also declined by more than 60% (C3: 73%, C4: 65%). The decline of these immune components tended to become flat after 3 treatment procedures, similar to the change of AQP4-IgG. The analysis of lymphocyte subsets revealed that the natural killer (NK) cells had a remarkable decrease in percent (before *vs* after: 13.85% *vs* 3.54%) and number (before *vs* after: 265 cells/μL *vs* 98 cells/μL), while the number of T cells and B cells rose significantly, with the total lymphocytes (T cells + B cells + NK cells) elevating from 1905 to 2753 cells/μL (**Table 2**). The proportion of the activated T cells (CD3+HLA-DR+) and activated Ts cells (CD3+CD8+HLA-DR+)/Ts decreased from 10.28% and 17.57% to 6.91% and 12.23%, respectively. The percent of regulatory T cells (Treg, CD3+CD4+CD25+CD127low+) had a mild drop (before *vs* after: 5.08% *vs* 4.6%), with a major decline of the natural Treg cells (CD45RA+CD3+CD4+CD25+CD127low+) (before *vs* after: 1.69 *vs* 1.16%). There were unapparent differences in the interferon-γ (IFN-γ) producing lymphocytes (PMA/ionomycin-stimulated lymphocyte function assay) before and after treatment. The concentration of pro-inflammatory cytokines including interleukin-8 (IL-8) and tumor necrosis factor-α (TNF-α) also reduced, with an insignificant change in IL-6 level (electrochemiluminescence method, Roche Diagnostics) (**Table 2**). The patient did not report any discomfort and no infection or thrombosis occurred during the therapy. Notably, her symptoms did not improve with the reduction of AQP4-IgG or other immune components until the end of the 5th session. She got a recovery from bilateral complete blindness to hand move, and the final EDSS score was assessed at 5 one week after the last session at the timing of discharge. *Mycophenolate mofetil (1500mg/d)* instead of *tacrolimus (3mg/d)* was given as the maintenance treatment afterward. She refused a repeated test for immunoglobulins, complement proteins, cytokine profiles, and lymphocyte subsets when discharged. Disability including paraplegia and visual disturbance further ameliorated (visual acuity: OS: 0.6, OD: 0.2), with the EDSS of 3, and no relapse or drug-related adverse event was reported in the next 6-month follow-up.

**Table 1:** The changes in the level of serum immunoglobulins and complement proteins (C3 and C4) before and after each session of immunoadsorption.

	IgA, g/L	IgG, g/L	IgM, g/L	C3, g/L	C4, g/L
**Before 1st session**	1.57	7.6	0.12	0.82	0.23
**After 1st session**	1.12	1.3	0.06	0.53	0.16
**Before 2nd session**	1.08	3.2	0.06	0.69	0.2
**After 2nd session**	0.91	0.4	0.05	0.47	0.15
**Before 3rd session**	1.15	1.6	0.06	0.67	0.22
**After 3rd session**	0.86	<0.3	<0.04	0.35	0.12
**Before 4th session**	0.99	1	0.05	0.48	0.16
**After 4th session**	0.8	<0.3	0.04	0.26	0.12
**Before 5th session**	0.97	0.9	0.04	0.44	0.14
**After 5th session**	0.74	<0.3	<0.04	0.22	0.08
**Reduction Rate, %**	52.9	>96.1	>66.7	73.2	65.2
**Reference Range**	0.82-4.53	7.51-15.6	0.46-3.04	0.65-1.39	0.16-0.38

**Table 2:** The changes of lymphocyte subsets and cytokines before and after treatment.

Lymphocyte subsets and cytokines	Before 1st session	After 5th session	Reference range
Total T cells (CD3+CD19-), %	66.09	74.04	50-84
Total T cell count (CD3+CD19-), cells/μL	1265	2048	955-2860
Total B cells (CD3-CD19+), %	19.58	21.95	5-18
Total B cell count (CD3-CD19+), cells/μL	375	607	90-560
Th cells (CD3+CD4+), %	41.03	43.93	27-51
Th cell count (CD3+CD4+), cells/μL	786	1215	550-1440
*Ts cells (CD3+CD8+), %	19.66	26.29	15-44
Ts cell count (CD3+CD8+), cells/μL	376	727	320-1250
NK cells (CD3-/CD16+CD56+), %	13.85	3.54	7-40
NK cell count (CD3-/CD16+CD56+), cells/μL	265	98	150-1100
T cells +B cells + NK cells, %	99.52	99.53	95-105
T cell +B cell + NK cell count, cells/μL	1905	2753	
Th/Ts	2.09	1.67	0.71-2.78
Th (CD3+CD4+CD28+)/Th, %	93.91	97.77	84.11-100.00
Tc (CD3+CD8+CD28+)/Ts, %	81.42	91.54	48.04-77.14
Activated T cells (CD3+HLA-DR+), %	10.28	6.91	9.04-25.62
Activated Ts cells (CD3+CD8+HLA-DR+)/Ts, %	17.57	12.23	20.73-60.23
Naïve Th cells (CD3+CD4+CD45RA+)/Th, %	35.8	30.34	36.41-57.07
Memory Th cells (CD3+CD4+CD45RO+)/Th, %	64.2	69.66	44.44-68.94
Treg (CD3+CD4+CD25+CD127low+), %	5.08	4.6	3.13-6.49
Natural Treg (CD45RA+CD3+CD4+CD25+CD127low+), %	1.69	1.16	2.07-4.55
Induced Treg (CD45RO+CD3+CD4+CD25+CD127low+), %	3.39	3.44	1.03-2.29
IFN-γ producing CD4+ T cells/Th, %	18.8	18.77	14.54-36.96
IFN-γ producing CD8+ T cells/Ts, %	29.49	25.72	34.93-87.95
IFN-γ producing NK cells/NK cells, %	72.25	65.88	61.2-92.65
IL-1β (pg/ml)	<5	<5	<5
IL-2R (U/ml)	582	378	223-710
IL-6 (pg/ml)	2.25	2	<7.0
IL-8 (pg/ml)	41.7	15.2	<62
IL-10 (pg/ml)	<5	<5	<9.1
TNF-α (pg/ml)	9.9	4.3	<8.1

*Ts cells (CD3+CD8+) included Tc (CD3+CD8+CD28+) and Ts (CD3+CD8+CD28-).

Th cell, helper T cell; Ts cell, suppressor T cell; Tc cell, cytotoxic T cell; NK cell, natural killer cell; Treg cell, regulatory T cell; IFN , interferon; IL-2R, interleukin-2 receptor; TNF, tumor necrosis factor.

## Discussion

We report a case of AQP4-IgG seropositive steroid-resistant NMOSD with severe relapse who profited from protein-A immunoadsorption. The rapid and sawtooth-like decrease of the complement proteins and antibody levels, especially AQP4-IgG, the declined pro-inflammatory mediators, as well as the subsequent clinical improvement suggest that immunoadsorption could be one of the rescue therapeutic options for severely affected NMOSD patients.

Protein-A immunoadsorption selectively eliminates immunoglobulins by filtering plasma through columns containing *Staphylococcus* cell wall–derived protein A. Each protein-A molecule has three potential binding sites for IgG, with a more potent affinity for it than IgM (11). Further, other circulating immune components including complement proteins and inflammatory cytokines can also be removed (12), although the underlying mechanisms remain to be fully elucidated. In line with these, we observed a sharp decrease in the serum level of AQP4-IgG and the biggest reduction rate (>96.1%) in (total-)IgG in our case, followed by IgM (>66.7%) and IgA (53%), with a drop of complement C3 and C4 simultaneously. Moreover, 3 sessions could reduce the IgG to below the detectable limit and the decline in immunoglobulin and complement protein levels tended to become flat, implying that 3 or 4 immunoadsorption procedures might be enough in treating antibody-mediated autoimmune diseases. In addition, the immunoglobulin depletion in the serum can cause an osmotic equilibration between extra- and intravascular space (13), probably leading to a reduction of pathogenic antibodies and other immune complexes e.g. complement proteins in the central nervous system (CNS) and finally minimizing the irreversible CNS damage. This redistribution was observed indirectly by a slight rebound of sero-immunoglobulins before every treatment and contributed to the sawtooth-like kinetics of antibody concentrations in the bloodstream (**Figure 3B**, **Table 1**).

Besides, as an IgG1-isotype antibody, AQP4-IgG could trigger the complement cascade and may cause complement-dependent cytotoxicity, which was observed by the vasculo-centric deposition of immunoglobulins and complement components in the acute lesions (14). Theoretically, complement depletion treatment may attenuate the CNS damage through the reduced formation of the membrane attack complex, which is implicated in astrocyte destruction and neuronal injury (15). Also, this therapy resulted in a significantly lower risk of relapse in patients with NMOSD (15). Generally, the complement system can be activated through 3 different pathways: the classical, lectin, and alternative pathways (16). Complement C4 component *via* the classical and/or lectin pathways and C3 *via* the alternative pathway are required in producing inflammatory mediators such as C3a and C4a and proceeding the complement cascade (16). In previous studies, the activation of complement C3 appeared to be positively associated with disease activity and neurological disability in patients with NMOSD (17, 18). Consistent with this, after 5 immunoadsorption procedures, the circulating levels of complement proteins including C3 and C4 in our case had significant removal rates of 73.2% and 65.2%, respectively, with the clinical improvement and a short relapse-free period thereafter. However, when compared to IgG, complement components seemed less vulnerable to be eliminated by immunoadsorption therapy alone (7). Besides, the activation of the complement system could also be possibly suppressed after the clearance of AQP4-IgG and therefore reduced binding to AQP4.

A marked increase of the T cell and B cell counts was observed after the therapy (**Table 2**), with a reduced proportion of the activated T (CD3+HLA-DR+) and Ts cells (CD3+CD8+HLA-DR+), implying a possibly improved immune system, which could be a downstream effect after the clearance of the pro-inflammatory mediators. In contrast, the percent and number of NK cells had a pronounced drop, which was also noted by previous studies as a potential biomarker candidate for acute-phase NMOSD (19, 20), although the underlying cause has been still unclear. A significantly increased level of Tregs (CD3+CD4+CD25+CD127low+) after immunoadsorption treatment in a previous study (21) was not found in our case. Also, the concentration of pro-inflammatory cytokines including IL-8 and TNF-α reduced, which may lower the disease activity, while an elevated IL-6 level during the acute phase, noted by previous researches (22, 23), was not observed here, either.

Generally, pulsed high-dose IVMP is widely used as the first-line treatment for acute exacerbations of NMOSD, with considerable benefit in most patients. Glucocorticoids act by inhibiting a series of inflammation processes through multiple mechanisms, including reducing the proinflammatory cytokines and suppressing the T-cell activation. However, steroid resistance is probably the summative effect of polymorphism of the glucocorticoid receptor (24), altered cytokines expression (25), etc., which compromises the anti-inflammatory activity of glucocorticoids, although the exact underlying causes are still not fully understood. Theoretically, protein-A immunoadsorption can rapidly decrease loads of the circulating pathogenic antibodies and pro-inflammatory mediators, possibly exerting a faster and potent anti-inflammatory effect than glucocorticoids or serving as adjuvant therapy to improve the sensitivity to steroids, especially for refractory cases with extensive and serious injuries.

It is noteworthy that although the pathogenic AQP4-IgG can induce a series of inflammatory cascades, causing damage to the CNS, the clinical improvement did not occur instantly with the reduction of the serum AQP4-IgG until the 5th session. This may be associated with delayed depletion of the immune complexes in the CNS and necessary time for neural repair. Moreover, although AQP4-IgG is much more concentrated in plasma than in CSF^73^, suggesting the peripheral origin and secondary entry to the CNS, the serum AQP4-IgG in our case persisted (1:32) while the symptoms remitted, implying that the circulating AQP4-IgG alone is insufficient to produce NMOSD lesions (14). It has also been supported by the previous observations that the serum AQP4-IgG might be present for years and will increase in concentration before attacks (3, 26). Nevertheless, it is one of the limitations that the data on IgA, IgG, IgM, C3 or C4, and cytokine levels, as well as the lymphocyte subsets 2 weeks after discharge, were missing due to the patient’s refusal.

Besides, the side effects and costs of a particular treatment should also be taken into account. In the previous studies (9, 27) on autoimmune encephalitis, the immunoadsorption therapy was almost well tolerated. The venous catheter-related adverse events should be drawn attention, albeit no thrombosis or patient-reported discomfort occurred in our case. Although this treatment, admittedly, has been still costly from the patient perspective, which is the major limitation for wide application, the potential benefits from the reduced neurologic impairment, accelerated clinical recovery, and the short length of hospital stay may outweigh its risks and costs in patients with severe NMOSD acute attack.

Nonetheless, after all, immunoadsorption is not a panacea for all the NMOSD attacks, for those non-responders after 5 procedures, more sessions seem feasible (28). Moreover, even if this treatment could not contribute to any remission in a short term, the patient could still benefit from the removal of pathogenic immune complexes, which may help provide a temporarily stable immunological seedbed for further neural repair and an improved outcome achieved by the subsequent long-term and slow-acting immunotherapies e.g. *mycophenolate mofetil (1500mg/d)* in our case.

## Conclusions

We demonstrated the changes in the serum level of AQP4-IgG, immunoglobulins, complement proteins (C3 and C4), and cytokine profiles as well as the alterations of lymphocyte subsets in a protein-A immunoadsorption treated case. Immunoadsorption can exert the anti-inflammatory effect *via* rapid clearance of the pathogenic antibodies and other immune components and could be one of the potential rescue therapies for steroid-resistant NMOSD patients with a severe disability.

## Data Availability Statement

The original contributions presented in the study are included in the article/supplementary material. Further inquiries can be directed to the corresponding authors.

## Ethics Statement

The studies involving human participants were reviewed and approved by Tongji hospital of Tongji medical college, Huazhong University of Science and Technology. The patients/participants provided their written informed consent to participate in this study. Written informed consent was obtained from the individual(s) for the publication of any potentially identifiable images or data included in this article.

## Author Contributions 

BC analyzed the data and wrote the manuscript. CQ, MC, H-HY, RT, and Y-HC were responsible for collecting the data. B-TB and D-ST cared for the patient, designed and revised the manuscript. All authors contributed to the article and approved the submitted version.

## Funding 

The study was supported by National Natural Science Foundation of China (Grants: 82071380, 81873743).

## Conflict of Interest

The authors declare that the research was conducted in the absence of any commercial or financial relationships that could be construed as a potential conflict of interest.

## References

[1] Wingerchuk DM Banwell B Bennett JL Cabre P Carroll W Chitnis T. International Consensus Diagnostic Criteria for Neuromyelitis Optica Spectrum Disorders. Neurology (2015) 85(2):177–89. 10.1212/WNL.0000000000001729 PMC4515040 26092914

[2] Jarius S Ruprecht K Wildemann B Kuempfel T Ringelstein M Geis C. Contrasting Disease Patterns in Seropositive and Seronegative Neuromyelitis Optica: A Multicentre Study of 175 Patients. J Neuroinflam (2012) 9:14. 10.1186/1742-2094-9-14 PMC3283476 22260418

[3] Jarius S Wildemann B. AQP4 Antibodies in Neuromyelitis Optica: Diagnostic and Pathogenetic Relevance. Nat Rev Neurol (2010) 6(7):383–92. 10.1038/nrneurol.2010.72 20639914

[4] Weinshenker BG Wingerchuk DM Vukusic S Linbo L Pittock SJ Lucchinetti CF. Neuromyelitis Optica IgG Predicts Relapse After Longitudinally Extensive Transverse Myelitis. Ann Neurol (2006) 59(3):566–9. 10.1002/ana.20770 16453327

[5] Kleiter I Gahlen A Borisow N Fischer K Wernecke KD Wegner B. Neuromyelitis Optica: Evaluation of 871 Attacks and 1,153 Treatment Courses. Ann Neurol (2016) 79(2):206–16. 10.1002/ana.24554 26537743

[6] Kleiter I Gahlen A Borisow N Fischer K Wernecke KD Hellwig K. Apheresis Therapies for NMOSD Attacks: A Retrospective Study of 207 Therapeutic Interventions. Neurol Neuroimmunol Neuroinflamm (2018) 5(6):e504. 10.1212/NXI.0000000000000504 30345331 PMC6192689

[7] Defendi F Malvezzi P Eskandary F Cesbron JY Rostaing L Böhmig GA. Effects of Immunoadsorption Combined With Membrane Filtration on Complement Markers - Results of a Randomized, Controlled, Crossover Study. Transpl Int (2019) 32(8):876–83. 10.1111/tri.13431 30901502

[8] Naganuma T Furusawa Y Hanaoka A Takemoto Y Uchida J. A Case of Anti-Aquaporin-4 Antibody-Positive Optic Neuritis Treated by Selective Immunoadsorption. Transfus Apher Sci (2021) 60(1):102969. 10.1016/j.transci.2020.102969 33268303

[9] Heine J Ly LT Lieker I Slowinski T Finke C Prüss H. Immunoadsorption or Plasma Exchange in the Treatment of Autoimmune Encephalitis: A Pilot Study. J Neurol (2016) 263(12):2395–402. 10.1007/s00415-016-8277-y 27604620

[10] Nishimura H Enokida H Sakamoto T Takahashi T Hayami H Nakagawa M. Immunoadsorption Plasmapheresis Treatment for the Recurrent Exacerbation of Neuromyelitis Optica Spectrum Disorder With a Fluctuating Anti-Aquaporin-4 Antibody Level. J Artif Organs (2018) 21(3):378–82. 10.1007/s10047-018-1044-3 29675599

[11] Howe RB Christie DJ. Protein A Immunoadsorption Treatment in Hematology: An Overview. J Clin Apheresis (1994) 9(1):31–2. 10.1002/jca.2920090109 8195110

[12] Oji S Nomura K. Immunoadsorption in Neurological Disorders. Transfus Apher Sci (2017) 56(5):671–6. 10.1016/j.transci.2017.08.013 28919008

[13] Klingel R Heibges A Fassbender C. Neurologic Diseases of the Central Nervous System With Pathophysiologically Relevant Autoantibodies–Perspectives for Immunoadsorption. Atheroscler Suppl (2013) 14(1):161–5. 10.1016/j.atherosclerosissup.2012.10.024 23357159

[14] Papadopoulos MC Verkman AS. Aquaporin 4 and Neuromyelitis Optica. Lancet Neurol (2012) 11(6):535–44. 10.1016/S1474-4422(12)70133-3 PMC3678971 22608667

[15] Pittock SJ Berthele A Fujihara K Kim HJ Levy M Palace J. Eculizumab in Aquaporin-4-Positive Neuromyelitis Optica Spectrum Disorde. N Engl J Med (2019) 381(7):614–25. 10.1056/NEJMoa1900866 31050279

[16] Ling M Murali M. Analysis of the Complement System in the Clinical Immunology Laborator. Clin Lab Med (2019) 39(4):579–90. 10.1016/j.cll.2019.07.006 31668271

[17] Nytrova P Potlukova E Kemlink D Woodhall M Horakova D Waters P. Complement Activation in Patients With Neuromyelitis Optica. J Neuroimmunol (2014) 274(1-2):185–91. 10.1016/j.jneuroim.2014.07.001 25109258

[18] Veszeli N Füst G Csuka D Trauninger A Bors L Rozsa C. A Systematic Analysis of the Complement Pathways in Patients With Neuromyelitis Optica Indicates Alteration But No Activation During Remission. Mol Immunol (2014) 57(2):200–9. 10.1016/j.molimm.2013.09.010 24172223

[19] Yandamuri SS Jiang R Sharma A Cotzomi E Zografou C Ma AK. High-Throughput Investigation of Molecular and Cellular Biomarkers in NMOSD. Neurology(R) Neuroimmunol Neuroinflam (2020) 7(5). 10.1212/NXI.0000000000000852 PMC7413712 32753407

[20] Ding J Zhu DS Hong RH Wu YF Li ZZ Zhou XJ. The Differential Expression of Natural Killer Cells in NMOSD and MS. J Clin Neurosci (2020) 71:9–14. 10.1016/j.jocn.2019.11.022 31864829

[21] Bulut D Creutzenberg G Mügge A. The Number of Regulatory T Cells Correlates With Hemodynamic Improvement in Patients With Inflammatory Dilated Cardiomyopathy After Immunoadsorption Therapy. Scand J Immunol (2013) 77(1):54–61. 10.1111/sji.12000 22998220

[22] Uzawa A Mori M Arai K Sato Y Hayakawa S Masuda S. Cytokine and Chemokine Profiles in Neuromyelitis Optica: Significance of Interleukin-6. Multiple Sclerosis (2010) 16(12):1443–52. 10.1177/1352458510379247 20739337

[23] Fujihara K Bennett JL de Seze J Haramura M Kleiter I Weinshenker BG. Interleukin-6 in Neuromyelitis Optica Spectrum Disorder Pathophysiology. Neurology(R) Neuroimmunol Neuroinflam (2020) 7(5). 10.1212/NXI.0000000000000841 PMC7455314 32820020

[24] Tantisira KG Lasky-Su J Harada M Murphy A Litonjua AA Himes BE. Genomewide Association Between GLCCI1 and Response to Glucocorticoid Therapy in Asthma. N Engl J Med (2011) 365(13):1173–83. 10.1056/NEJMoa0911353 PMC3667396 21991891

[25] Leung DY Martin RJ Szefler SJ Sher ER Ying S Kay AB. Dysregulation of Interleukin 4, Interleukin 5, and Interferon Gamma Gene Expression in Steroid-Resistant Asthma. J Exp Med (1995) 181(1):33–40. 10.1084/jem.181.1.33 7807013 PMC2191836

[26] Nishiyama S Ito T Misu T Takahashi T Kikuchi A Suzuki N. A Case of NMO Seropositive for Aquaporin-4 Antibody More Than 10 Years Before Onset. Neurology (2009) 72(22):1960–1. 10.1212/WNL.0b013e3181a82621 19487655

[27] Dogan Onugoren M Golombeck KS Bien C Abu-Tair M Brand M Bulla-Hellwig M. Immunoadsorption Therapy in Autoimmune Encephalitides. Neurology(R) Neuroimmunol Neuroinflam (2016) 3(2):e207. 10.1212/NXI.0000000000000207 PMC4772911 26977423

[28] Kobayashi M Nanri K Taguchi T Ishiko T Yoshida M Yoshikawa N. Immunoadsorption Therapy for Neuromyelitis Optica Spectrum Disorders Long After the Acute Phase. J Clin Apher (2015) 30(1):43–5. 10.1002/jca.21324 24802352

